# GPX7 marks fibroblast-associated stromal–innate immune crosstalk in ulcerative colitis

**DOI:** 10.3389/fimmu.2026.1856998

**Published:** 2026-05-22

**Authors:** Jingui He, Rongrong Chen, Shaofan Qiu, Peimin Li, Xin Gao

**Affiliations:** 1 Department of Digestive Endoscopy, Fuzhou University Affiliated Provincial Hospital, Fuzhou, Fujian, China; 2 Department of Gastroenterology, Fuzhou University Affiliated Provincial Hospital, Fuzhou, Fujian, China; 3 Shengli Clinical Medical College of Fujian Medical University, Fuzhou, Fujian, China; 4 Department of Gastrointestinal Surgery, The Second Hospital of Hebei Medical University, Shijiazhuang, China; 5 Department of Gastroenterology, The Fifth Clinical Medical College of Shanxi Medical University, Taiyuan, Shanxi, China

**Keywords:** fibroblast–macrophage crosstalk, GPX7, immunometabolism, innate immunity, stromal remodeling, ulcerative colitis

## Abstract

**Background:**

Ulcerative colitis is sustained by stromal remodeling and innate immune activation. Whether serine-glycine-one-carbon metabolism contributes to fibroblast–macrophage crosstalk in ulcerative colitis remains unclear.

**Methods:**

We integrated two-sample Mendelian randomization, cross-cohort colonic transcriptomics, and single-cell RNA sequencing to prioritize serine-glycine-one-carbon–related candidates in ulcerative colitis, followed by qPCR validation in an infliximab-stratified cohort and a fibroblast–macrophage Transwell co-culture model. Signature-reversal analysis was used to nominate candidate modulators, whereas docking and molecular dynamics were applied as hypothesis-generating structural analyses.

**Results:**

GPX7 emerged as the most consistently supported candidate, with concordant mucosal upregulation and orthogonal cis-pQTL support. GPX7-high mucosa was enriched for complement, chemokine, one-carbon metabolism, and extracellular matrix programs. Single-cell analyses localized GPX7 predominantly to a fibroblast state characterized by increased CXCL12 and MMP2 expression, strengthened intercellular communication, and elevated HIF1A activity, consistent with stromal–innate immune coupling. In an independent clinical cohort, GPX7 expression was higher in biopsies from infliximab non-responders than responders. In a fibroblast–macrophage Transwell system, co-culture amplified macrophage inflammatory transcripts and fibroblast remodeling-associated transcripts under LPS stimulation, and QL-X-138 attenuated these responses in an exploratory perturbation setting. Docking and molecular dynamics analyses provided preliminary structural plausibility for QL-X-138 as a candidate modulator of the GPX7-associated program.

**Conclusion:**

GPX7 marks a genetically supported fibroblast-associated stromal–innate immune crosstalk state in ulcerative colitis and supports further mechanistic investigation of stromal remodeling and innate inflammatory signaling.

## 1 Introduction

Ulcerative colitis (UC) is a chronic, relapsing inflammatory disease of the colonic mucosa with substantial clinical and molecular heterogeneity (1–3). Despite advances in treat-to-target management and biologic or small-molecule therapy, primary non-response and secondary loss of response remain major clinical challenges (4, 5). These limitations highlight unresolved disease drivers and the need for biologically grounded patient stratification (3, 6). A persistent interaction occurs between epithelial damage, innate immune activation, recruitment of inflammatory cells, activation of fibroblasts, and ECM remodeling in UC. These activities reinforce a maladaptive inflammatory-repair loop (7, 8).

Metabolic rewiring is becoming an increasingly important determinant of inflammatory states. Nonetheless, what the exact circuits are that link mucosal immune activation to stromal remodeling in UC remains poorly resolved (9, 10). Metabolism of serine-glycine-one-carbon (SGOC) is a promising candidate, as it ties together nucleotide synthesis, methyl donor availability, glutathione-dependent redox buffering, and transsulfuration, and thus modifies inflammatory output, cellular stress responses, and tissue repair (11–13). In immune systems, SGOC flux fuels effector programs. For example, in T cells, SGOC flux supports serine-dependent entry into one-carbon metabolism during T-cell expansion. Similarly, in macrophages, SGOC flux mediates serine-glycine-glutathione (SGG) support of macrophage IL-1β production (14). In parallel, one-carbon metabolism can directly sustain fibroblast collagen production downstream of profibrotic cues (15), suggesting a route by which immunometabolic stress can be translated into ECM remodeling. In UC, recent work has linked serine metabolism to epithelial and immune cell function in colitis (16), but a genetically anchored, cell-resolved framework connecting SGOC biology to innate immune-stromal circuitry is still lacking.

Here, we integrated Mendelian randomization (MR), cross-cohort mucosal transcriptomics, single-cell mapping, and targeted experimental validation to prioritize SGOC-related candidates associated with UC. We prioritized candidates using cis-eQTL-based MR against UC GWAS summary statistics with independent replication (17), then evaluated the reproducibility of prioritized signals across colonic transcriptomic cohorts. We further integrated orthogonal molecular-QTL evidence and MR-based mediation (18), mapped the prioritized candidate within a colonic single-cell atlas to resolve cell-state localization, transcriptional control, and intercellular communication, and used human biopsy qPCR together with a fibroblast–macrophage Transwell co-culture model to assess clinical concordance and stromal–innate immune crosstalk. This strategy identified glutathione peroxidase 7 (GPX7) as a genetically supported candidate associated with immunometabolic fibroblast activation and innate immune signaling in UC.

## 2 Materials and methods

### 2.1 Public resources and study design

This study integrated (i) transcriptomic profiles from public Gene Expression Omnibus (GEO) cohorts, (ii) blood cis-eQTL summary statistics for genetic instruments, (iii) UC GWAS summary statistics for outcomes, (iv) methylation- and protein-QTL resources for mediation and orthogonal support, (v) colonic single-cell RNA-seq for cell-type localization and downstream mechanistic analyses, and (vi) targeted qPCR validation in human samples and a fibroblast–macrophage co-culture system. GEO datasets used for model training and testing included GSE107499 (UC mucosal biopsies), GSE47908 (UC and control mucosa), and GSE87466 (UC and control mucosa). A detailed summary of all data resources, analytical roles, and processing filters is provided in **Table 1**.

**Table 1:** Data resources and processing overview.

Data type	Resource	Tissue/population	Role in study	Key filters/processing
SGOC gene set	MSigDB (C2/KEGG-related), GeneCards, Harmonizome	NA	Define SGOC-related universe	Merge + HGNC symbol standardization; deduplicate
eQTL (discovery)	eQTLGen (blood cis-eQTL)	Whole blood	MR exposures (gene expression)	P<5×10^-8^; clump 10,000 kb r²<0.1; F>10; Steiger filtering
eQTL (replication)	GTEx v10 whole blood	Whole blood	MR replication	Same thresholds as discovery
Outcome GWAS	FinnGen R12 UC	European	MR outcome	Harmonization; IVW primary; Egger/WM sensitivity
pQTL	deCODE plasma proteomics summary data	Icelandic plasma	Proteome-level support	cis ±1Mb; P<5×10^-8^; clump 10,000 kb r²<0.1
mQTL	GoDMC/mQTL portals	Blood DNAm	Two-step MR mediation	instruments P<5×10^-8^; clump 10,000 kb r²<0.1; delta-method SE
Bulk transcriptomics	GSE107499	Colon mucosa	ML training; UC-only clustering	Scaling using training mean/SD; ML benchmarking
Bulk transcriptomics	GSE47908	Colon mucosa	External validation	Same preprocessing
Bulk transcriptomics	GSE87466	Colon mucosa	External validation	Same preprocessing
Anti-TNF baseline	GSE16879	Colon mucosa	Therapy response association	Baseline responders vs non-responders
scRNA-seq	GSE214695 (6 HC, 6 UC)	Colon mucosa	Cell-type localization + fibro analysis	Seurat QC+Harmony; clustering res=0.5; pseudo-bulk logCPM; pctExpr>0

### 2.2 Identification of SGOC-related genes

SGOC-related genes were obtained from three complementary resources: (i) curated pathway gene sets from Molecular Signatures Database (MSigDB) (KEGG-related collections), (ii) keyword-based retrieval from GeneCards (restricted to protein-coding genes), and (iii) pathway gene sets from Harmonizome. Gene symbols were standardized to HUGO Gene Nomenclature Committee (HGNC) nomenclature and merged after removing duplicates, yielding 1,978 unique SGOC-related genes (**Supplementary Table 1**).

### 2.3 Blood cis-eQTL instrument selection (discovery and replication)

Cis-eQTL summary statistics were obtained from the eQTLGen Consortium (whole-blood cis-eQTL meta-analysis (19)). Genetic instruments were selected as genome-wide significant cis-eQTLs (P < 5×10^-8^), followed by linkage disequilibrium (LD) clumping (10,000 kb window; r² < 0.1). After filtering, genes with ≥1 eligible cis-eQTL were retained for MR screening, and then intersected with the SGOC gene set to define the candidate SGOC cis-eQTL targets.

For independent replication, Genotype-Tissue Expression (GTEx) v10 whole blood eQTL summary statistics were obtained from the GTEx Portal (20). The same instrument selection (P < 5×10^-8^; 10,000 kb; r² < 0.1), harmonization, and filtering steps were applied. Because these discovery and replication instruments were derived from whole blood, they may not fully capture colonic mucosal or fibroblast-specific regulatory effects. We therefore used blood cis-eQTL MR for genetic prioritization rather than definitive tissue-specific causal inference, and interpreted the MR results together with colonic transcriptomic and single-cell evidence.

### 2.4 Two-sample mendelian randomization

UC GWAS summary statistics were obtained from FinnGen release 12. Two-sample MR was conducted using the TwoSampleMR framework (21). For each gene, cis-eQTL instruments were harmonized with UC GWAS data using allele-alignment procedures that remove ambiguous strand cases when appropriate. Instrument strength was evaluated using per-variant R² and F-statistics; variants with F ≤ 10 were excluded. Directionality was assessed by Steiger filtering to remove variants explaining more variance in the outcome than in the exposure.

Primary causal estimates were obtained using inverse-variance weighted (IVW) MR, with MR-Egger and weighted median as sensitivity estimators. Effect sizes were reported as odds ratios (ORs) with 95% confidence intervals.

### 2.5 Proteome-level support using deCODE cis-pQTL instruments

Cis-pQTL summary data were downloaded from deCODE genetics summary data (Icelandic population plasma proteomics (22)). Cis-pQTLs were defined as SNPs within ±1 Mb of the coding gene, P < 5×10^-8^, LD clumping 10,000 kb and r² < 0.1, consistent with the eQTL MR settings.

### 2.6 Two-step MR mediation

For mediation, methylation QTL (mQTL) summary statistics were obtained from public mQTL resources (GoDMC/mQTL portals (23)). A two-step MR framework was used to quantify the indirect effect of methylation (exposure) on UC (outcome) through gene expression (mediator). The indirect effect was estimated as a*b, where a denotes the MR effect of methylation on expression and b denotes the MR effect of expression on UC. Standard errors were computed using the delta method. The direct effect was estimated as c’ = c − a*b, where c denotes the total MR effect of methylation on UC.

### 2.7 Machine learning model development and evaluation

A multi-algorithm machine learning (ML) benchmarking pipeline was implemented in R (glmnet, randomForestSRC, gbm, xgboost, e1071, mboost, caret, pROC, ComplexHeatmap). Models were trained on the training cohort and tested in external cohorts. Expression matrices were standardized using training-set mean/SD (train-derived scaling applied to test data). Multiple algorithms and two-stage combinations (feature selection + classifier) were evaluated; models with <5 retained features were excluded. Performance was quantified by AUC (ROC), including training cross-validation and external-cohort testing AUCs. Model interpretation used SHapley Additive exPlanations (SHAP) values, and the top contributors were summarized. Feature scaling parameters were estimated only in the training cohort and then applied to external validation cohorts. Model development and feature selection were performed within the training framework, whereas external cohorts were held out for final testing to reduce data leakage. The ML component was used for biological prioritization and interpretability rather than clinical diagnostic deployment; therefore, calibration and decision-curve analyses were not pursued.

### 2.8 Consensus clustering using five SHAP-highlighted genes

Using UC samples from the training cohort, unsupervised subtyping was performed by ConsensusClusterPlus with k = 2 to define two stable clusters (C1/C2). Differential expression between clusters was tested by limma, and five-gene heatmaps were visualized after z-scoring by gene.

### 2.9 Gene set enrichment analysis

Gene Set Enrichment Analysis (GSEA) was performed with 10,000 permutations using MSigDB C2 curated gene sets; enriched pathways were summarized by normalized enrichment score (NES) and reported at FDR q < 0.1.

### 2.10 Immune/stromal deconvolution

Immune cell abundance was estimated using MCPcounter (24, 25), and global stromal/immune components were quantified using ESTIMATE scores (26).

### 2.11 Anti-TNF response cohort

Baseline (pre-treatment) colonic transcriptomes from anti-TNF–treated UC patients were obtained from GSE16879. Patients were stratified as responders versus non-responders at baseline as provided by the dataset annotation.

### 2.12 Clinical qPCR validation in infliximab response strata

To assess the clinical relevance of GPX7 in treatment response, GPX7 mRNA expression was quantified by qPCR in colonic mucosal biopsies from patients with ulcerative colitis (UC) stratified by infliximab (IFX) response. The cohort comprised responders (n = 15) and patients with primary non-response (n = 15). This analysis was designed to determine whether the GPX7-associated expression pattern identified in the public datasets was concordant in an independent clinical cohort. All clinical samples were obtained from Fujian Provincial Hospital Affiliated to Fuzhou University, and the study protocol was conducted in accordance with the principles of the Declaration of Helsinki. The study was approved by the Ethics Committee of Fujian Provincial Hospital Affiliated to Fuzhou University (Approval No. K2026-04-005). Written informed consent was obtained from all participants prior to sample collection.

### 2.13 THP-1 macrophage differentiation and fibroblast–macrophage co-culture with QL-X-138 perturbation

Human THP-1 monocytes (RRID: CVCL_0006) and CCD-18Co intestinal fibroblasts (RRID: CVCL_2379) were obtained from the National Collection of Authenticated Cell Cultures, Chinese Academy of Sciences. THP-1 monocytes were differentiated into adherent macrophage-like cells by incubation with 100 ng/mL phorbol 12-myristate 13-acetate (PMA; Sigma-Aldrich, St. Louis, MO, USA) for 48 h, followed by a 24 h resting period in PMA-free complete medium. For stromal–innate immune crosstalk experiments, a Transwell co-culture system (0.4 μm pore size; Corning, NY, USA) was established with CCD-18Co intestinal fibroblasts seeded in the lower chamber and THP-1-derived macrophages seeded in the upper inserts. Cells were exposed to three conditions: (1) LPS-stimulated monoculture controls (1 μg/mL LPS); (2) LPS-stimulated fibroblast–macrophage co-culture; and (3) LPS-stimulated co-culture treated with 10 μM QL-X-138. After 24 h, macrophages and fibroblasts were harvested separately for RNA extraction. GPX7, IL-1β, TNF, and IL6 expression was measured in THP-1-derived macrophages, whereas GPX7, CXCL12, and MMP2 expression was measured in CCD-18Co fibroblasts. CCD-18Co fibroblasts and THP-1-derived macrophages were used as standardized human cell-line models for mechanistic exploration. These models may not recapitulate the epigenetic imprinting, inflammatory history, or activated baseline of UC patient-derived primary fibroblasts and primary myeloid cells.

### 2.14 RNA extraction and quantitative real-time PCR

Total RNA was extracted from clinical mucosal biopsies and from THP-1-derived macrophages and CCD-18Co fibroblasts harvested from the Transwell system using TRIzol™ Reagent (Invitrogen, Carlsbad, CA, USA) according to the manufacturer’s instructions. RNA concentration and purity were assessed by spectrophotometry (NanoDrop™ 2000, Thermo Fisher Scientific). Reverse transcription was performed using the PrimeScript™ RT Reagent Kit (Takara Bio, Shiga, Japan) to synthesize complementary DNA (cDNA). Real-time quantitative PCR was performed using SYBR^®^ Green PCR Master Mix (Applied Biosystems, Foster City, CA, USA) on a QuantStudio™ 5 Real-Time PCR System (Applied Biosystems). In the clinical cohort, GPX7 expression was quantified. In the co-culture experiments, GPX7, IL-1β, TNF, and IL6 were assessed in THP-1-derived macrophages, whereas GPX7, CXCL12, and MMP2 were assessed in CCD-18Co fibroblasts. Relative gene expression was normalized to ACTB (β-actin) as the endogenous control. The specific primer sequences used for amplification are listed in **Table 2**.

**Table 2:** Primer sequences used for qRT-PCR.

Gene	Forward primer (5’-3’)	Reverse primer (5’-3’)
*GPX7*	CGACTTCAAGGCGGTCAACATC	TCGGTAGTGCTGGTCTGTGAAG
*CXCL12*	CTCAACACTCCAAACTGTGCCC	CTCCAGGTACTCCTGAATCCAC
*IL1B(IL-1β)*	CCACAGACCTTCCAGGAGAATG	GTGCAGTTCAGTGATCGTACAGG
*TNF*	CCTCTCTCTAATCAGCCCTCTG	GAGGACCTGGGAGTAGATGAG
*IL6*	ACTCACCTCTTCAGAACGAATTG	CCATCTTTGGAAGGTTCAGGTTG
*MMP2*	TACAGGATCATTGGCTACACACC	GGTCACATCGCTCCAGACT
*ACTB(β-actin)*	CACCATTGGCAATGAGCGGTTC	AGGTCTTTGCGGATGTCCACGT

### 2.15 Single-cell RNA-seq processing and GPX7 localization

Colonic single-cell RNA sequencing (scRNA-seq) data were obtained from GSE214695 (27), including healthy controls (n=6) and UC (n=6) (CD samples present but not used for HC vs UC comparisons). Raw matrices were processed using the Seurat package: cells with low feature counts were removed; mitochondrial fraction was computed and high-mitochondrial-content cells excluded. Following log-normalization of the data, variable features were identified, and the data were scaled prior to principal component analysis (PCA). Batch effects across samples were corrected using Harmony (28), followed by neighbor graph construction, clustering (resolution = 0.5), and Uniform Manifold Approximation and Projection (UMAP) and t-distributed Stochastic Neighbor Embedding (t-SNE) visualization. Cell types were annotated using canonical marker panels and cluster markers.

### 2.16 Pseudo-bulk logCPM and pctExpr

For each cell type, sample-level pseudo-bulk was computed by aggregating counts across cells within each sample and converting to log2-CPM (logCPM). Detectable expression for pctExpr was defined as expression > 0 at the single-cell level; pctExpr was computed as the fraction of cells with detectable expression per sample and cell type.

### 2.17 Fibroblast GPX7 stratification and virtual knockout

Within UC fibroblasts, we classified cells as GPX7-positive (GPX7_POS) or GPX7-negative (GPX7_NEG) based on detectable GPX7 expression (expression > 0). First, differential expression analysis was performed between these two groups. The resulting gene lists were subsequently subjected to GSEA and Gene Ontology (GO) analysis, using the same statistical thresholds established earlier. In parallel, we conducted a virtual knockout analysis using scTenifoldKnk (29) to infer the regulatory consequences of GPX7 perturbation. The enrichment results from this analysis were summarized using identical parameters to ensure direct comparability with the differential expression-based findings. Because this binary detectable-expression definition may be influenced by scRNA-seq dropout, GPX7_POS/GPX7_NEG analyses were interpreted as fibroblast-state association analyses rather than strict fibroblast subtype definitions.

### 2.18 Cell–cell communication analysis

Cell–cell communication was inferred using the ligand–receptor interaction probability framework CellChat. Subsequently, we compared the communication strength and the patterns of incoming and outgoing signaling between the GPX7_POS and GPX7_NEG fibroblast states.

### 2.19 Transcription factor activity analysis

Per-cell TF activity scores were treated as a quantitative matrix (cells × TFs) generated by a predefined regulon/TF-target–based inference pipeline (details reflected in the analysis code used to generate the TF activity matrix). Differential transcription factor (TF) activity between GPX7_POS and GPX7_NEG fibroblasts was assessed using the same statistical framework applied to other single-cell analyses. For each TF, we reported the average log2 fold-change (avg_log2FC) and the Benjamini-Hochberg (BH) adjusted p-value.

### 2.20 In silico compound nomination using LINCS L1000 resources

To prioritize candidate small molecules capable of reversing the GPX7-associated transcriptional state, we performed signature-based perturbagen screening using LINCS L1000 resources. A gene signature consisting of up- and down-regulated genes (adjusted P < 0.05) was submitted to CLUE/Connectivity Map (CMap, LINCS L1000) (30) and L1000CDS2 (31) in reverse mode to obtain ranked candidate compounds. Compounds supported by both platforms (intersection hits) were prioritized for downstream structure-based evaluation.

### 2.21 Molecular docking

The crystal structure of GPX7 was obtained from the Protein Data Bank and used as the target protein. Ligand 3D structures were downloaded from PubChem and energy-minimized under the MMFF94 force field prior to docking. The target protein was prepared in PyMOL v2.5.5 by removing crystallographic water molecules, salt ions, and non-relevant small molecules.

Molecular docking was performed using AutoDock Vina v1.2.3 (32, 33). A docking grid box was defined to encompass the entire protein, consistent with a blind docking setup. The prepared protein and ligands were converted to the required PDBQT format using ADFRsuite v1.03. Docking was conducted with an exhaustiveness of 32, while other parameters were kept at default settings. The top-ranked pose (lowest predicted binding energy) was selected as the representative binding conformation. Docking poses were visualized and analyzed using PyMOL v2.5.5 and LigPlot v2.2.9.

### 2.22 Molecular dynamics simulation

All-atom MD simulations were performed using AMBER 24. The protein was described using the ff14SB force field (34), and ligands were parameterized using GAFF2 with AM1-BCC charges (35). Each complex was solvated in an explicit TIP3P water box with a 10 Å buffer and neutralized by adding counterions.

Energy minimization was carried out using 2,500 steps of steepest descent followed by 2,500 steps of conjugate gradient. The system was heated from 0 K to 298.15 K over 200 ps, followed by equilibration for 500 ps in the NVT ensemble and 500 ps in the NPT ensemble. Production simulations were then conducted for 50 ns in the NPT ensemble.

A nonbonded cutoff of 10 Å was used. Long-range electrostatics were treated with the particle mesh Ewald (PME) method, and bonds involving hydrogens were constrained using SHAKE. Temperature was controlled using a Langevin thermostat (collision frequency 2 ps^-1^), and pressure was maintained at 1 atm. The integration time step was 2 fs, and coordinates were saved every 10 ps for subsequent analyses.

### 2.23 Trajectory analyses and MM/GBSA binding free energy

The analytical techniques employed to study various trajectories involved a complex RMSD, ligand RMSD, hydrogen bond counts, RMSF, radius of gyration (Rg), and solvent-accessible surface area (SASA). The Molecular Mechanics/Generalized Born Surface Area (MM/GBSA) method was used to estimate binding free energies (ΔGbind). MM/GBSA calculations were performed on frames extracted from the 40–50 ns window of the production trajectory. The generalized Born model was specified to igb = 2 (36), and the nonpolar solvation contribution was computed as GSA = 0.0072 × SASA. Entropy contributions were not included. Per-residue energy decomposition was performed to identify key residues contributing to binding.

### 2.24 Statistical analysis

All statistical analyses were performed using R software (version 4.3.1) and Python. Unless otherwise stated, all statistical tests were two-tailed, and a P-value < 0.05 was considered statistically significant. Continuous variables, including qPCR measurements from clinical samples and cell experiments, were compared between two groups using the non-parametric Wilcoxon rank-sum test. For multiple comparisons, P-values were adjusted using the Benjamini-Hochberg method to control the false discovery rate. Correlations between gene expression signatures were assessed using Spearman’s rank correlation analysis. In Mendelian randomization, causal estimates were derived using the Inverse-Variance Weighted (IVW) method, with instrument strength validated via F-statistics (F > 10). Data from molecular dynamics simulations are presented as mean ± standard deviation (SD).

## 3 Results

### 3.1 Genome-wide MR prioritizes SGOC-related genes associated with UC

We curated a comprehensive SGOC-related gene set (1,978 genes; **Supplementary Table 1**) and performed a two-sample MR screen using blood cis-eQTL instruments against UC GWAS summary statistics. In the discovery stage (eQTLGen), IVW MR identified 182 SGOC-related genes showing nominal evidence of association with UC risk (**Supplementary Table 2**). These candidates were then evaluated in an independent whole-blood eQTL resource (GTEx v10), and 26 genes retained nominal support in replication, defining the final prioritized set for downstream analyses (**Supplementary Table 3**).

### 3.2 Cross-cohort transcriptomic prioritization highlights a core GPX7-centered program

**Figure 1:**
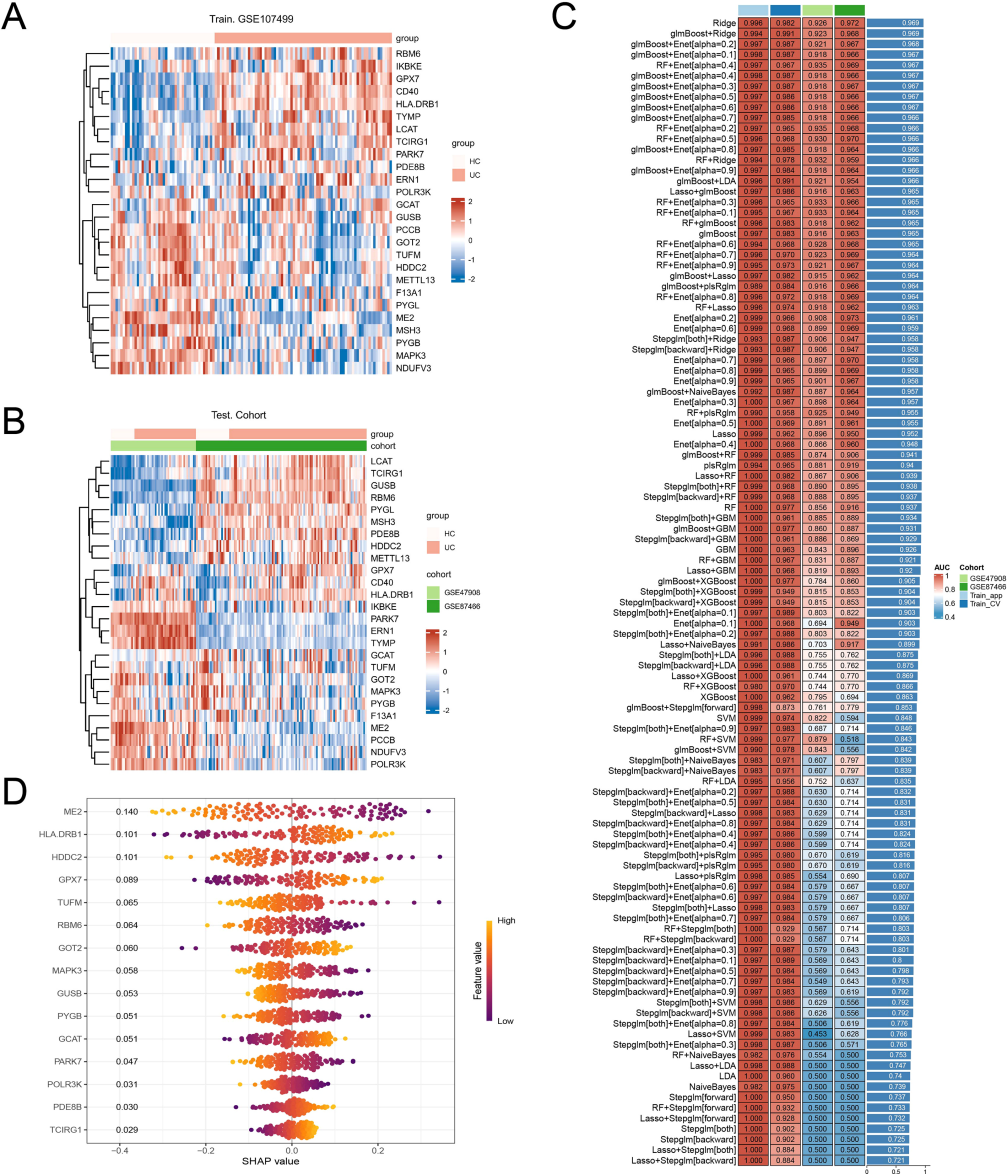
A 26-gene SGOC-linked program and cross-cohort prioritization. **(A)** Heatmap of the 26 replicated genes in the training cohort (GSE107499). Samples are annotated by group (UC vs control definition used in the training matrix). **(B)** Heatmap of the same 26 genes in external validation cohorts (GSE47908 and GSE87466). **(C)** AUC heatmap summarizing model performance across training (apparent and cross-validation) and external cohorts. Ridge regression shows consistently strong generalization. **(D)** SHAP summary plot showing the top features contributing to the best-performing model; five genes (ME2, HLA-DRB1, HDDC2, TUFM, GPX7) were selected for biological stratification.

Using the 26 replicated genes, we benchmarked multiple learning algorithms and two-stage combinations across one training and two external validation cohorts. The 26-gene expression patterns captured reproducible UC-associated structure across datasets (**Figures 1A, B**). Across model families, Ridge regression showed strong generalizability (**Figure 1C**). SHAP interpretation highlighted a compact set of dominant contributors—ME2, HLA-DRB1, HDDC2, TUFM, and GPX7 (**Figure 1D**)—which were carried forward not as a diagnostic signature, but as a tractable axis for mechanistic stratification.

### 3.3 Five-gene consensus clustering reveals an immunometabolic-ECM program

**Figure 2:**
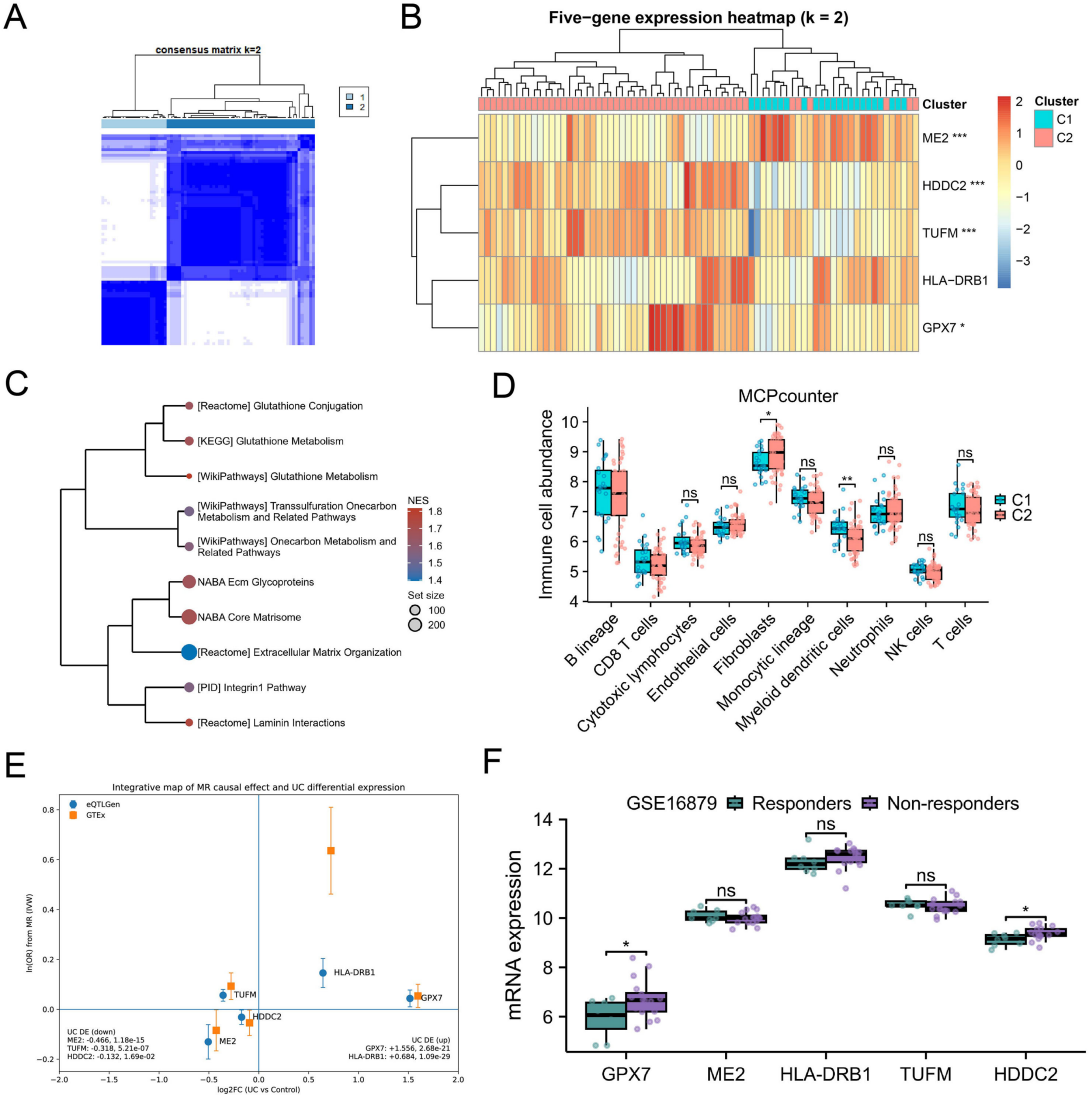
Five-gene consensus subtypes link immunometabolism, ECM remodeling, and immune context; GPX7 prioritized. **(A)** Consensus clustering matrix (ConsensusClusterPlus) supporting k = 2. **(B)** Five-gene expression heatmap (z-scored by gene) with cluster annotation (C1/C2). Stars indicate BH-FDR-adjusted significance for C2 vs C1 (* <0.05; ** <0.01; *** <0.001). **(C)** GSEA of C2 vs C1 (10,000 permutations; MSigDB C2 curated), highlighting glutathione metabolism, one-carbon/transsulfuration programs, and ECM pathways (FDR q < 0.1). **(D)** MCPcounter immune cell abundance comparing C1 vs C2; Wilcoxon test with BH-FDR across cell types (ns, not significant). **(E)** Integrative plot of MR causal estimates (eQTLGen discovery and GTEx replication where available) against UC differential expression; GPX7 shows concordant multi-layer evidence and is the only one among the five with supporting cis-pQTL analysis in deCODE under the stated filters. **(F)** Baseline expression of five genes in anti-TNF cohort (GSE16879; responders vs non-responders).

We conducted consensus clustering (k=2) on the five genes highlighted by SHAP in the UC samples of the training cohort to connect ML signals to biology in an interpretable way. Clustering revealed two stable subtypes (C1 and C2; **Figures 2A, B**). The C2 subtype showed stronger enrichment of SGOC-adjacent programs including those involved in glutathione metabolism and one-carbon/transsulfuration pathways along with ECM organization/remodeling signatures (**Figure 2C**). The five-gene axis appears to capture a higher-order metabolic–redox–matrix state connected to SGOC biology. The analysis of immune deconvolution further highlighted differences in microenvironment between the subtypes (**Figure 2D**). These differences were in accordance with coordinated metabolic and stromal remodeling.

### 3.4 Multi-modal nomination of GPX7 for mechanistic follow-up

We next integrated MR effect directions with UC case–control expression changes for the five SHAP-highlighted genes (**Figure 2E**). GPX7 and HLA-DRB1 showed concordant risk-increasing patterns, with ORs > 1 and higher expression in UC. In contrast, ME2 and HDDC2 displayed a coherent protective direction, with ORs < 1 accompanied by reduced expression in UC. TUFM showed discordant directionality between MR and expression, indicating that not all predictive features map onto a simple causal–expression alignment. To evaluate protein-level evidence, we queried cis-pQTL summary data using the same instrument-selection and LD-independence framework; only GPX7 retained eligible cis-pQTL instruments, whereas the other four genes did not have usable cis-pQTL evidence after filtering (**Figure 2E**).

In an independent anti-TNF baseline cohort (GSE16879), GPX7 expression differed between responders and non-responders prior to therapy (**Figure 2F**), suggesting exploratory relevance to anti-TNF response biology. Collectively, these observations prioritized GPX7 for deeper multi-scale characterization.

### 3.5 MR mediation suggests an epigenetic-expression link for GPX7 in UC

**Figure 3:**
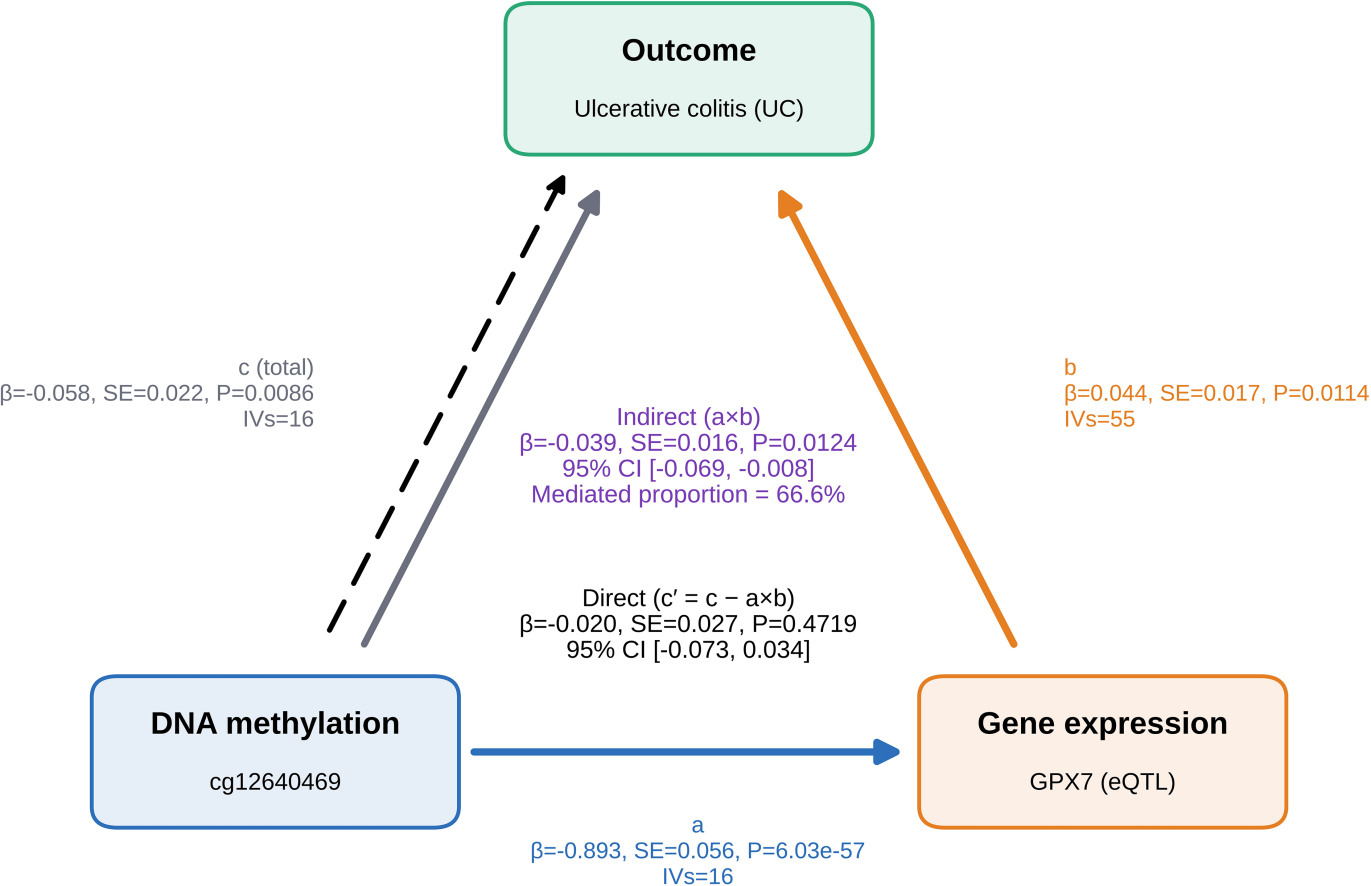
Two-step MR mediation links methylation, GPX7 expression, and UC risk. Schematic and results of two-step MR mediation: methylation → GPX7 expression → UC. Indirect (ab) and direct (c’) effects were estimated with delta-method standard errors. Instruments used P < 5×10^-8^ and LD clumping 10,000 kb/r² < 0.1; weak instruments (F ≤ 10) were excluded. mQTL sources were GoDMC/mQTL portals.

Two-step MR mediation supported a regulatory hierarchy. We found that DNA methylation at cg12640469 was associated with lower GPX7 expression and lower genetically predicted UC risk. Conversely, loss of this epigenetic constraint was associated with higher GPX7 expression and increased UC risk. Mediation analysis indicated that the methylation-to-expression pathway accounted for 66.6% of the total effect, supporting a model in which GPX7 is an important intermediate between local epigenetic regulation and disease-linked stromal activation (**Figure 3**).

### 3.6 GPX7-high mucosa is associated with innate immune and stromal activation

**Figure 4:**
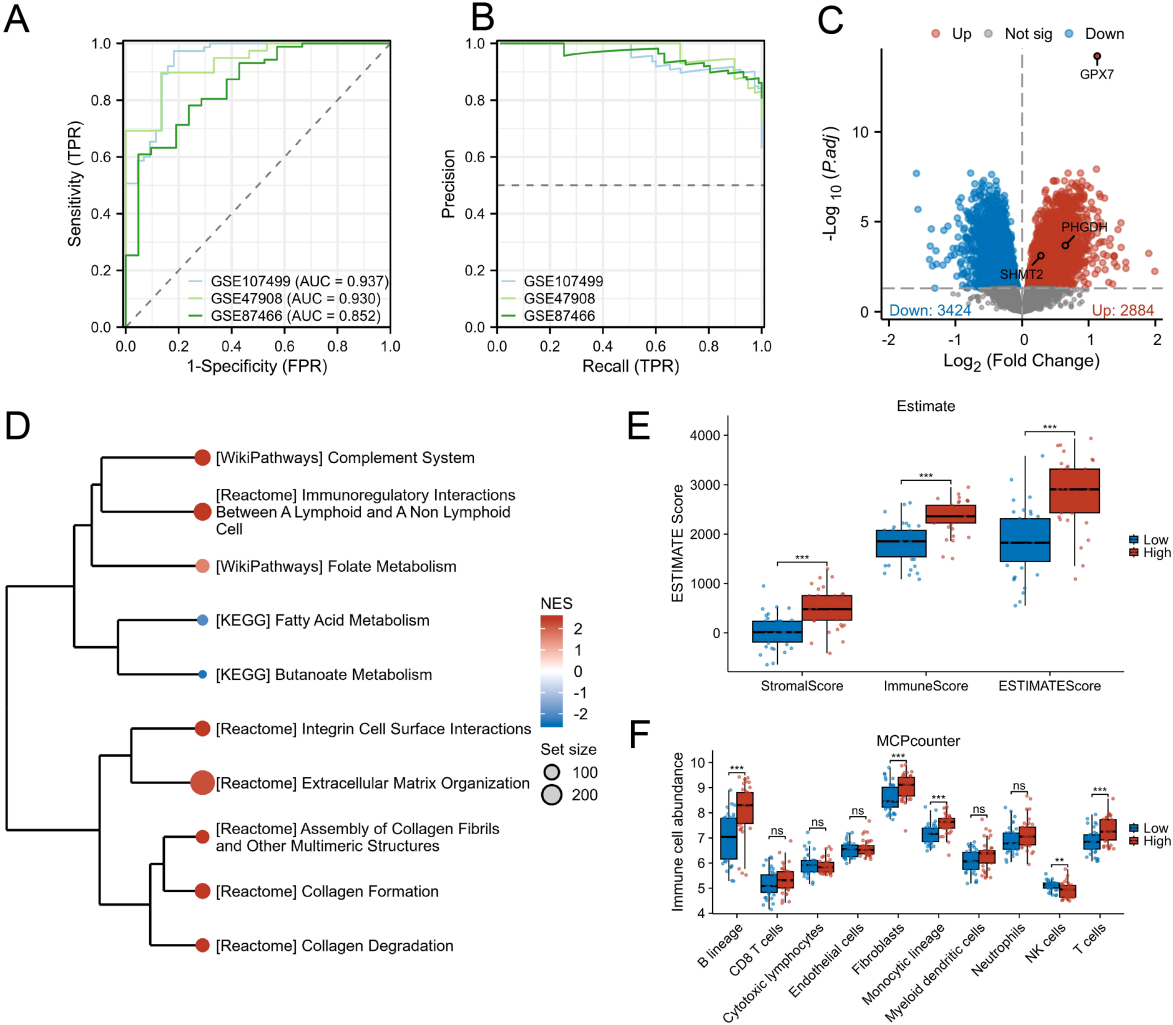
GPX7-high mucosa associates with innate immune-stromal activation. **(A)** ROC curves for GPX7-only classifier across cohorts. **(B)** Precision–recall curves across cohorts. **(C)** Volcano plot of UC vs control differential expression highlighting GPX7 and representative SGOC-related genes. **(D)** GSEA in GPX7-high vs GPX7-low samples showing complement/immune interaction, folate/one-carbon metabolism, and ECM modules. **(E)** ESTIMATE scores (stromal, immune, total) comparing GPX7-high vs GPX7-low; Wilcoxon with BH-FDR. **(F)** MCPcounter immune abundance comparing GPX7-high vs GPX7-low; Wilcoxon with BH-FDR.

Across cohorts, higher GPX7 expression reproducibly tracked with UC-related transcriptomic remodeling (**Figures 4A–C**). Pathway analysis in GPX7-high samples revealed enrichment of complement and immune interaction modules, folate and one-carbon metabolism, and ECM programs (**Figure 4D**). Microenvironment scores further supported elevated immune and stromal components in GPX7-high samples (**Figure 4E**), accompanied by coherent shifts in inferred immune cell abundance (**Figure 4F**).

### 3.7 Cell-type localization of GPX7 in colonic single-cell atlases highlights cycling and fibroblast compartments in UC

**Figure 5:**
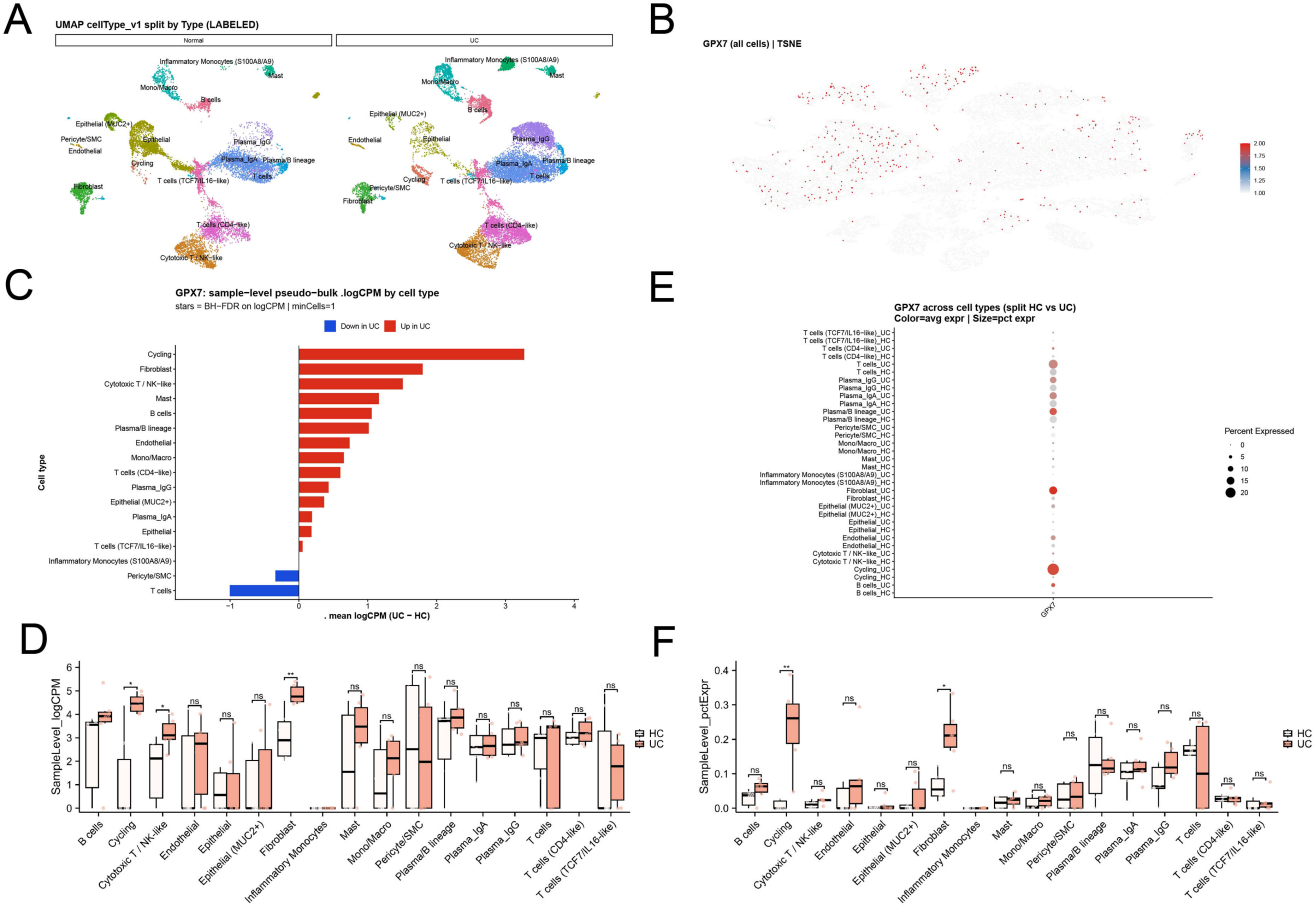
Single-cell localization of GPX7 in UC colonic mucosa. **(A)** UMAP split by phenotype (HC vs UC) with annotated cell types (Seurat + Harmony integration). **(B)** tSNE feature map showing GPX7 expression across all cells. **(C)** Sample-level pseudo-bulk logCPM difference (UC − HC) by cell type; stars indicate BH-FDR across cell types. **(D)** Boxplots of sample-level logCPM by cell type (UC vs HC). **(E)** Dot plot showing GPX7 average expression and percent expressed across cell types (split by HC vs UC). **(F)** Boxplots of sample-level pctExpr (fraction of cells with GPX7 expression > 0) by cell type (UC vs HC). scRNA-seq dataset: GSE214695 (6 HC, 6 UC).

We integrated colonic scRNA-seq profiles from healthy controls and UC and annotated major epithelial, stromal and immune compartments (**Figure 5A**). Quality control metrics and cell-type annotation are detailed in **Supplementary Figure 1**. Mapping GPX7 across the unified embedding indicated that GPX7 positive cells were not uniformly distributed, consistent with compartment- and state-specific expression (**Figure 5B**).

Pseudo-bulk expression analyses across cell types showed that the UC-associated increase in GPX7 was most pronounced in cycling cells and fibroblasts, with a smaller increase in cytotoxic T/NK-like cells (**Figure 5C**). This pattern was supported at the sample level: GPX7 logCPM was significantly elevated in UC in cycling, cytotoxic T/NK-like, and fibroblast compartments, whereas most other lineages showed non-significant differences (**Figure 5D**).

Expression coverage analyses further indicated that UC-associated changes were driven not only by higher average expression but also by a larger fraction of expressing cells. Dot plots of average expression and percent expressed highlighted the strongest shifts in cycling cells and fibroblasts (**Figure 5E**), and sample-level pctExpr was significantly increased in UC specifically for cycling and fibroblast compartments (**Figure 5F**).

Notably, fibroblasts constitute the principal effectors of mucosal extracellular matrix (ECM) production and remodeling. The preferential enrichment of GPX7 in fibroblast and cycling states in UC therefore provides a cellular anchor for the ECM-associated programs observed in bulk-derived stratification analyses earlier in the study. In addition, fibroblast-focused genetic evidence supported the same risk direction: GTEx v10 fibroblast cis-eQTL–based MR for GPX7 showed a risk-increasing effect (OR > 1, P < 0.05; **Figure 2E**), consistent with a fibroblast-centered mechanism.

### 3.8 GPX7_POS fibroblasts exhibit profibrotic and innate inflammatory rewiring

**Figure 6:**
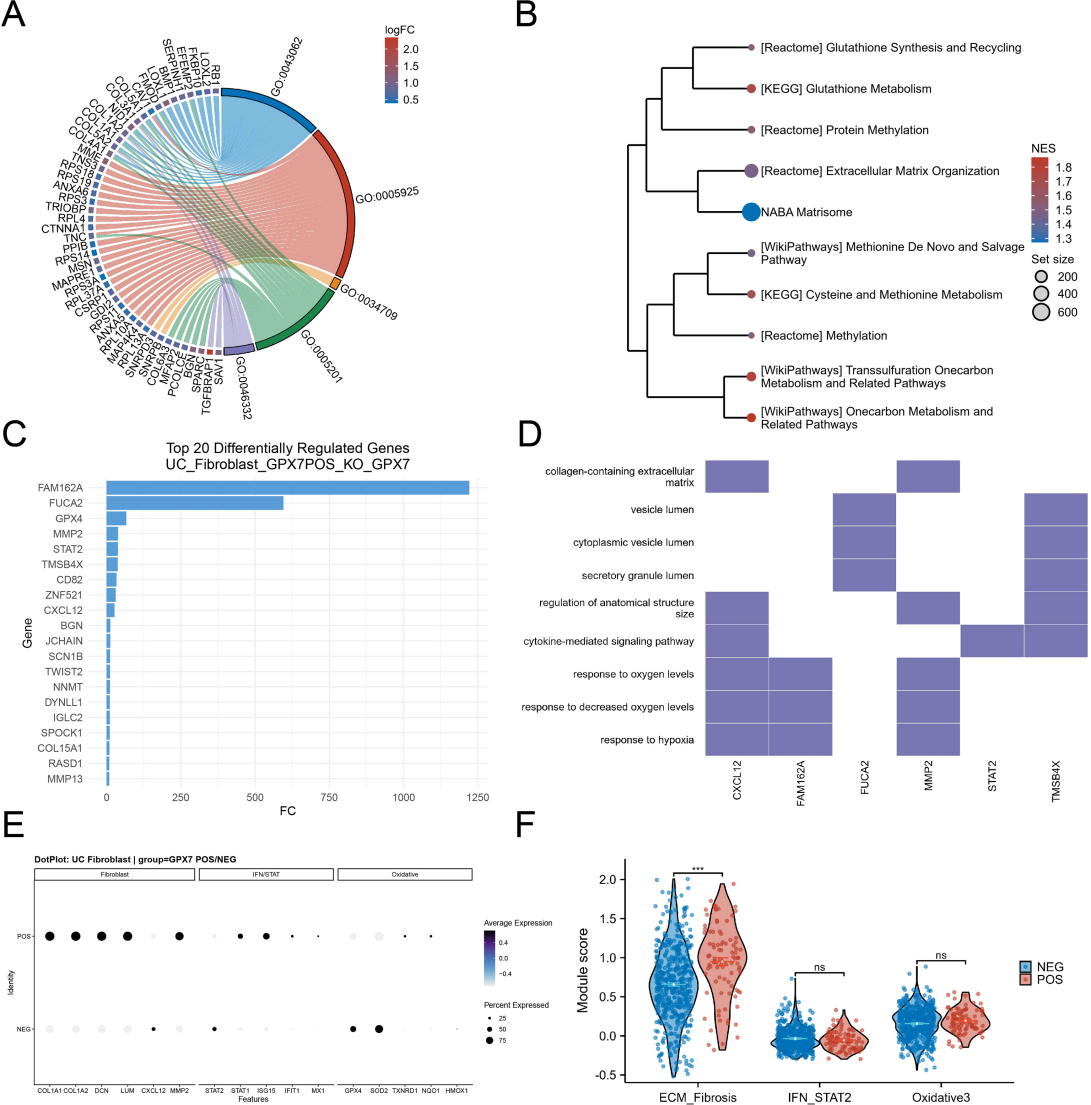
GPX7-positive UC fibroblasts show ECM/SGOC-related remodeling and innate inflammatory rewiring. **(A–F)** UC fibroblasts stratified into GPX7_POS vs GPX7_NEG (detectable expression >0) with downstream differential genes from scTenifoldKnk, functional enrichment (GSEA/GO), and module scoring (ECM/fibrosis, IFN/STAT, oxidative programs). scTenifoldKnk virtual knockout framework.

Focusing on UC fibroblasts, we stratified cells into GPX7_POS and GPX7_NEG states based on detectable GPX7 expression and performed differential expression analysis and pathway analyses (**Supplementary Table 4**). Differential expression between GPX7_POS and GPX7_NEG fibroblasts revealed an ECM- and adhesion-linked signature, with key stromal/ECM-associated genes contributing to the leading functional terms (**Figure 6A**). Consistent with the SGOC-adjacent biology highlighted in our bulk analyses, pathway enrichment further showed coordinated upregulation of glutathione synthesis/recycling and glutathione metabolism, together with cysteine–methionine/one-carbon and methylation-related pathways, alongside strong enrichment of extracellular matrix organization and matrisome programs (**Figure 6B**). To identify the upstream transcriptional drivers orchestrating this state, we inferred transcription factor (TF) activities based on regulon expression.

The analysis highlighted HIF1A and MEF2A as differentially active regulators in GPX7_POS fibroblasts. Increased HIF1A activity (avg_log2FC = 2.55, adjusted P = 0.020) provides a putative regulatory context for the hypoxia-response signatures observed in pathway analysis (**Supplementary Table 5**). Similarly, elevated MEF2A activity (avg_log2FC = 1.50, adjusted P = 0.027) is consistent with, rather than proving, a stromal remodeling-associated state.

To interrogate the regulatory consequences of GPX7 perturbation within this activated fibroblast state, we next performed an in silico GPX7 knockout using scTenifoldKnk in GPX7_POS UC fibroblasts. The perturbation analysis prioritized the downstream targets, including FAM162A, FUCA2, GPX4, MMP2, STAT2, CXCL12, BGN, TWIST2, NNMT and ECM-associated components (**Figure 6C**). Functional mapping of representative high-confidence outputs indicated convergence on biologically coherent processes, including collagen-containing extracellular matrix, vesicle/secretory lumen terms, cytokine-mediated signaling, and oxygen/hypoxia-response categories (**Figure 6D**).

Marker-level visualization supported the state assignment and its functional interpretation: GPX7_POS fibroblasts showed higher expression and/or broader detection of canonical fibroblast/ECM features (for example COL1A1/COL1A2, DCN, LUM) and remodeling/chemokine mediators (for example MMP2 and CXCL12) compared with GPX7_NEG fibroblasts (**Figure 6E**). Module scoring demonstrated a selective increase in an ECM/Fibrosis program in GPX7_POS fibroblasts, whereas IFN/STAT2 and an oxidative stress module were not significantly different between states (**Figure 6F**).

### 3.9 GPX7_POS fibroblasts define a high-communication stromal state with strengthened signaling exchange

**Figure 7:**
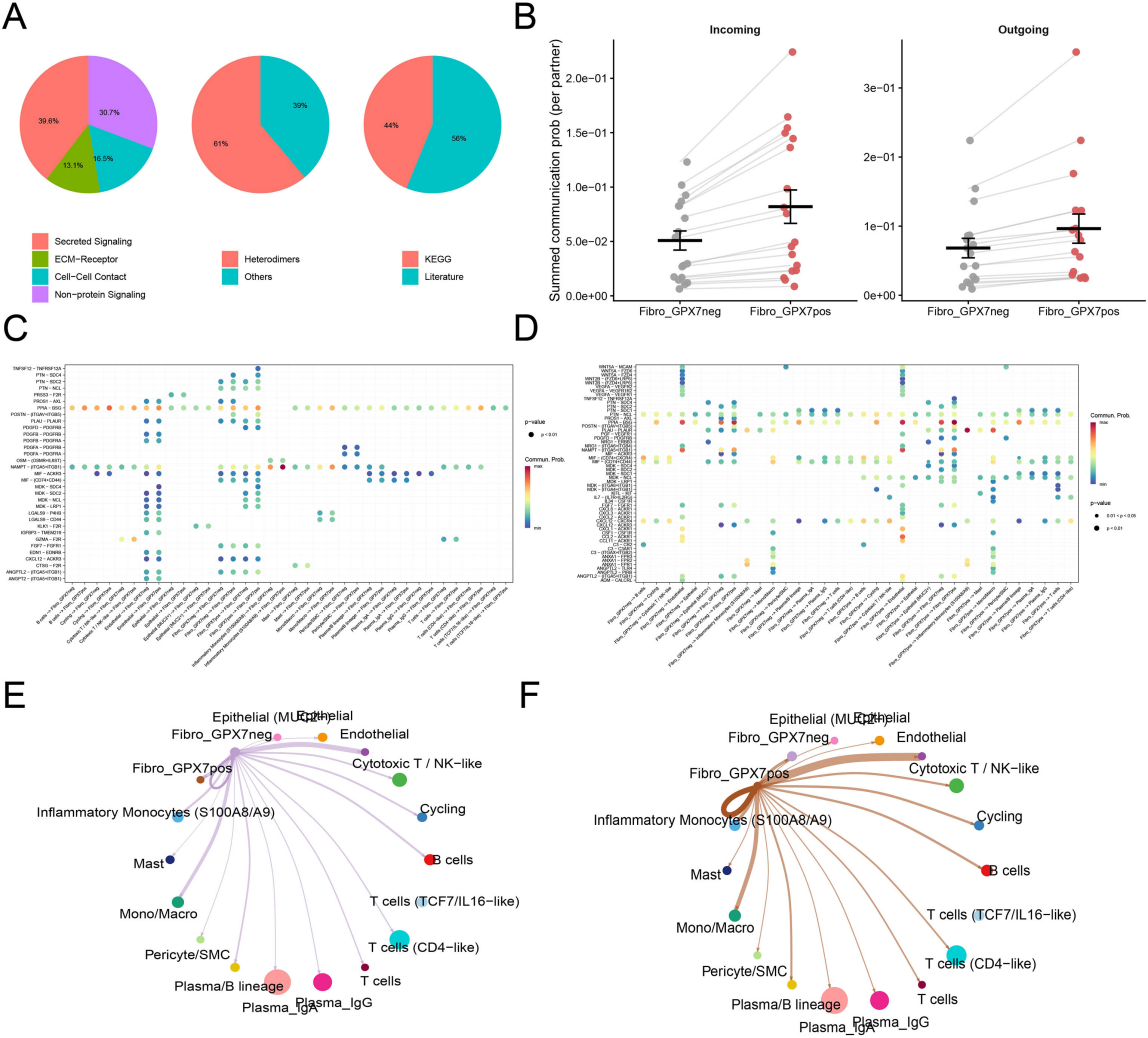
Cell–cell communication shifts associated with GPX7-positive fibroblast state. **(A)** Composition of inferred interaction categories. **(B)** Summed incoming/outgoing communication probability comparing GPX7_NEG vs GPX7_POS fibroblast states. **(C, D)** Bubble plots of significant ligand–receptor interactions (communication probability and P values). **(E, F)** Network visualization of fibroblast-centered outgoing communication differences. Cell–cell communication inferred using CellChat.

Because stromal remodeling is frequently reinforced by multicellular signaling circuits, we used CellChat to quantify ligand–receptor communication differences between GPX7_POS and GPX7_NEG fibroblast states. The inferred interaction landscape was dominated by secreted signaling, with additional contributions from non-protein signaling, cell–cell contact, and ECM–receptor interactions (**Figure 7A**). At a global level, GPX7_POS fibroblasts exhibited significantly higher incoming and outgoing communication strength than GPX7_NEG fibroblasts across partners (**Figure 7B**), supporting a model in which GPX7 positivity corresponds to a more interactive stromal state.

Differential interaction matrices indicated that this increase was distributed across multiple ligand–receptor axes (**Figures 7C, D**). Representative high-probability interactions encompassed ECM/adhesion and growth-factor modules (for example, integrin- and PDGFR-linked interactions), chemokine signaling (including CXCL12-associated axes), and inflammatory cytokine-related signaling, collectively consistent with enhanced stromal–immune and stromal–epithelial cross-talk in the GPX7_POS state (**Figures 7C, D**). Network visualizations further summarized these effects: compared with GPX7_NEG fibroblasts, GPX7_POS fibroblasts showed broadly stronger weighted connections to multiple tissue compartments, including epithelial, endothelial and diverse immune populations (**Figures 7E, F**).

### 3.10 Signature-reversal screening and structure-based analyses nominate QL-X-138 as an exploratory candidate modulator of the GPX7-associated program

**Figure 8:**
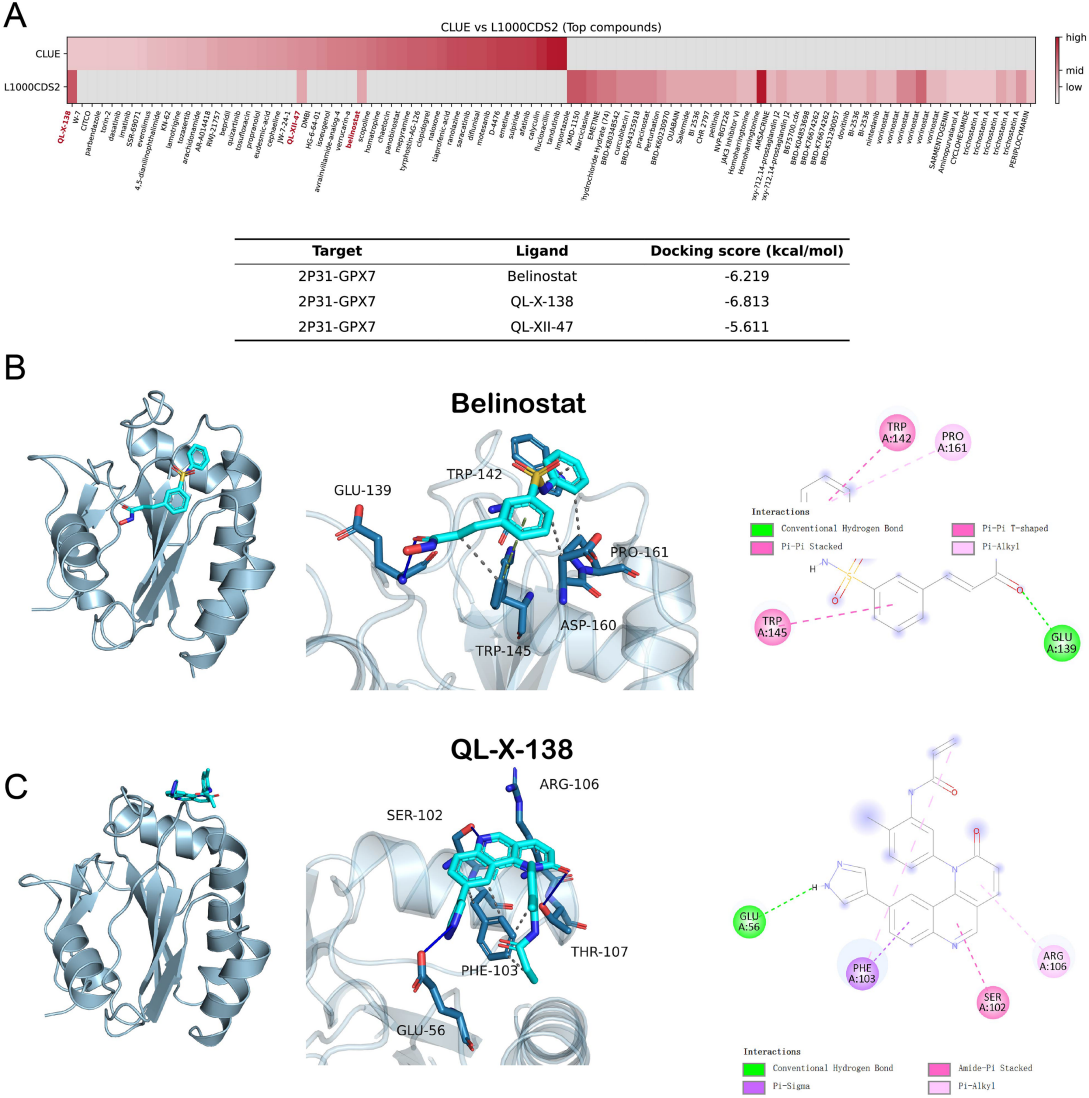
Computational prioritization of candidate modulators associated with the GPX7 program. **(A)** Heatmap visualization of connectivity scores from CLUE (CMap) and L1000CDS2 analysis. Columns represent ranked compounds; the intersection analysis highlights Belinostat, QL-X-138, and QL-XII-47 as convergent candidates capable of reversing the disease signature. **(B, C)** Predicted binding modes of Belinostat **(B)** and QL-X-138 **(C)** within the GPX7 binding pocket (PDB: 2P31). Left: 3D structural representations showing the ligand (cyan sticks) and key interacting residues (blue sticks). Right: 2D interaction diagrams illustrating hydrogen bonds (green/dashed lines) and hydrophobic/*π*–stacking interactions (pink/purple dashed lines). Belinostat occupies the pocket primarily via interactions with TRP142, TRP145, and GLU139, while QL-X-138 is anchored by PHE103 and ARG106.

To identify small molecules capable of reverting the GPX7-associated transcriptional program, we leveraged a signature-reversal strategy using LINCS L1000 perturbational profiles. Intersection analysis of top-ranked candidates from CLUE (CMap) and L1000CDS2 nominated three convergent hits—Belinostat, QL-X-138, and QL-XII-47—predicted to counteract the disease state (**Figure 8A**).

Structural evaluation via molecular docking against the GPX7 crystal structure (PDB: 2P31) indicated favorable binding feasibility for all candidates. Notably, QL-X-138 exhibited the strongest predicted affinity (Docking score = −6.81 kcal/mol), followed by Belinostat (−6.22 kcal/mol). QL-XII-47 showed a relatively lower binding score (−5.61 kcal/mol) and was predicted to engage a peripheral site involving LYS42 and GLN71 (**Supplementary Figure 2**); therefore, it was not prioritized for computationally expensive dynamic validation.

Focusing on the top two candidates, binding mode analysis revealed that Belinostat and QL-X-138 occupy a core pocket region but rely on distinct interaction networks. Belinostat is stabilized by hydrogen bonding with GLU139 and *π*-stacking interactions with TRP142/TRP145 (**Figure 8B**). In contrast, QL-X-138 is anchored primarily through hydrophobic and *π*-stacking interactions with PHE103 and ARG106 (**Figure 8C**). Based on their superior ranking and structural plausibility, these two compounds were advanced for molecular dynamics (MD) simulations.

### 3.11 MD simulations suggest a more stable equilibrated binding state for QL-X-138 than Belinostat

**Figure 9:**
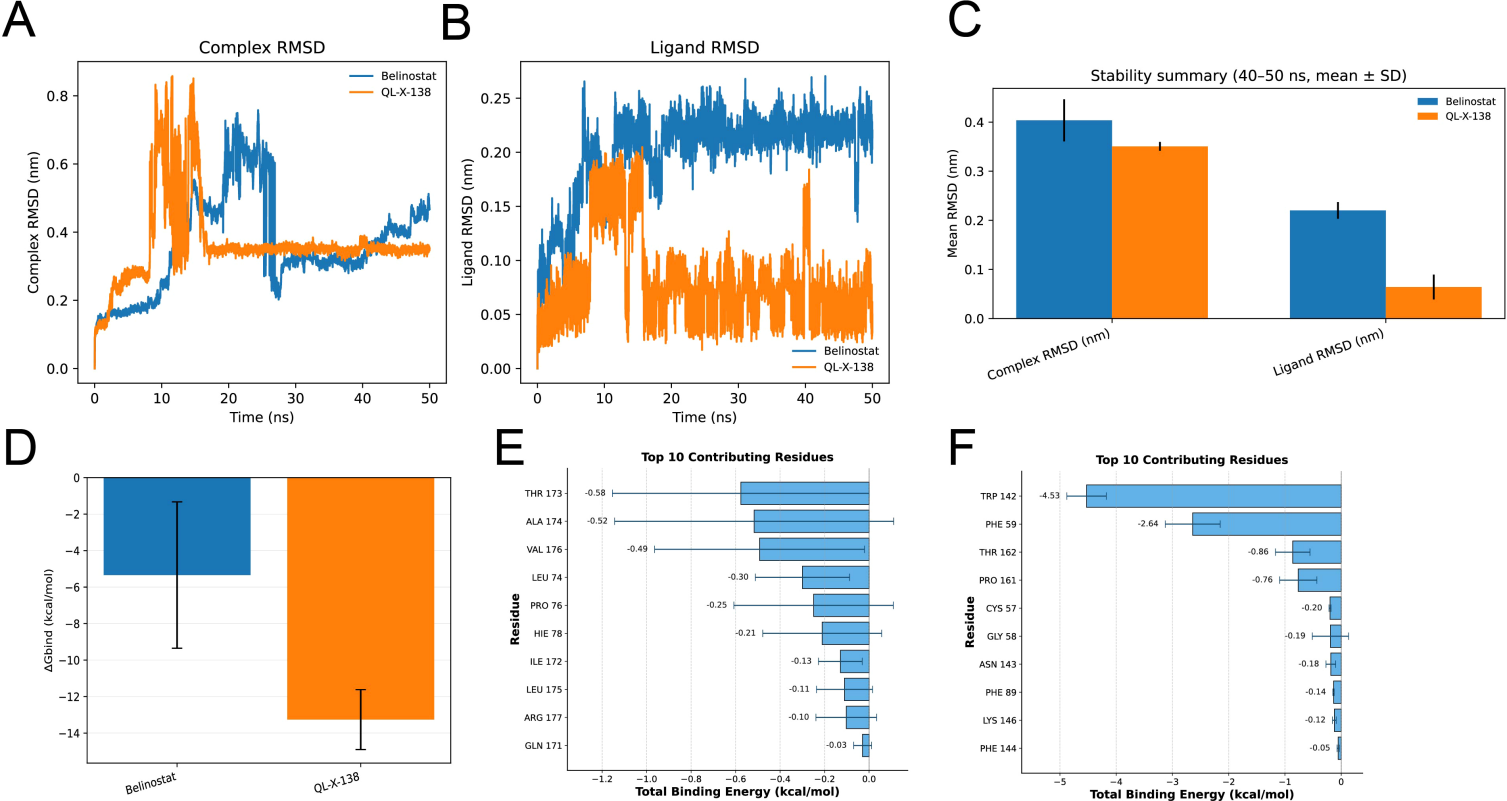
Molecular dynamics simulation and binding free-energy analyses of GPX7–ligand complexes. **(A, B)** Root-mean-square deviation (RMSD) trajectories of the GPX7–ligand complex **(A)** and the ligand **(B)** over a 50 ns simulation. Blue: Belinostat; Orange: QL-X-138. QL-X-138 undergoes an early conformational rearrangement followed by a lower-fluctuation equilibrium phase, whereas Belinostat remains more mobile over the simulation. **(C)** Mean complex RMSD and ligand RMSD calculated during the equilibrium phase (40–50 ns). Error bars represent standard deviation. **(D)** Binding free energies (ΔGbind) estimated using the MM/GBSA method. QL-X-138 shows a more negative ΔGbind than Belinostat. **(E, F)** Per-residue energy decomposition analysis identifying major amino acids contributing to ligand binding for Belinostat **(E)** and QL-X-138 **(F)**. Belinostat binding shows distributed and relatively modest residue contributions, whereas QL-X-138 exhibits a more concentrated energetic pattern dominated by TRP142 and supported by PHE59, THR162, and PRO161.

To rigorously evaluate the temporal stability of the prioritized complexes, we performed 50 ns MD simulations. The GPX7–Belinostat complex remained comparatively unstable throughout the trajectory, with complex RMSD rising after the early phase and continuing to fluctuate thereafter (**Figure 9A**). Its ligand RMSD also remained relatively high during the later phase, indicating persistent ligand mobility within the binding region (**Figure 9B**). Detailed structural parameter analysis further showed fluctuating radius of gyration (Rg) and solvent-accessible surface area (SASA) profiles for Belinostat, consistent with an unstable trajectory (**Supplementary Figure 3**).

In contrast, the GPX7–QL-X-138 complex underwent an early conformational rearrangement during approximately 8–15 ns, after which both the complex and the ligand entered a lower-fluctuation regime (**Figures 9A, B**). The equilibrium-phase summary over 40–50 ns further confirmed a slightly lower mean complex RMSD and a markedly lower mean ligand RMSD for QL-X-138 than for Belinostat (**Figure 9C**), consistent with tighter positional stabilization of the ligand after equilibration. In parallel, the complex Rg stabilized rapidly after this transition (**Supplementary Figure 4**), supporting the formation of a compact and stable binding state.

This dynamic stabilization was accompanied by more favorable binding energetics. MM/GBSA analysis showed that QL-X-138 exhibited a substantially more negative binding free energy than Belinostat (ΔGbind = −13.26 ± 1.64 kcal/mol vs. −5.34 ± 4.01 kcal/mol; **Figure 9D**). Per-residue energy decomposition revealed that Belinostat binding was supported by several modest contributors, including THR173, ALA174, and VAL176 (**Figure 9E**), whereas QL-X-138 displayed a more concentrated energetic pattern dominated by TRP142 and PHE59, with additional contributions from THR162 and PRO161 (**Figure 9F**). Together, these simulations suggest that QL-X-138 may form a more stable equilibrated binding state than Belinostat under the selected simulation settings. However, these findings remain preliminary and do not demonstrate direct GPX7 target engagement.

### 3.12 Clinical and co-culture validation supports GPX7-associated stromal–innate immune crosstalk

**Figure 10:**
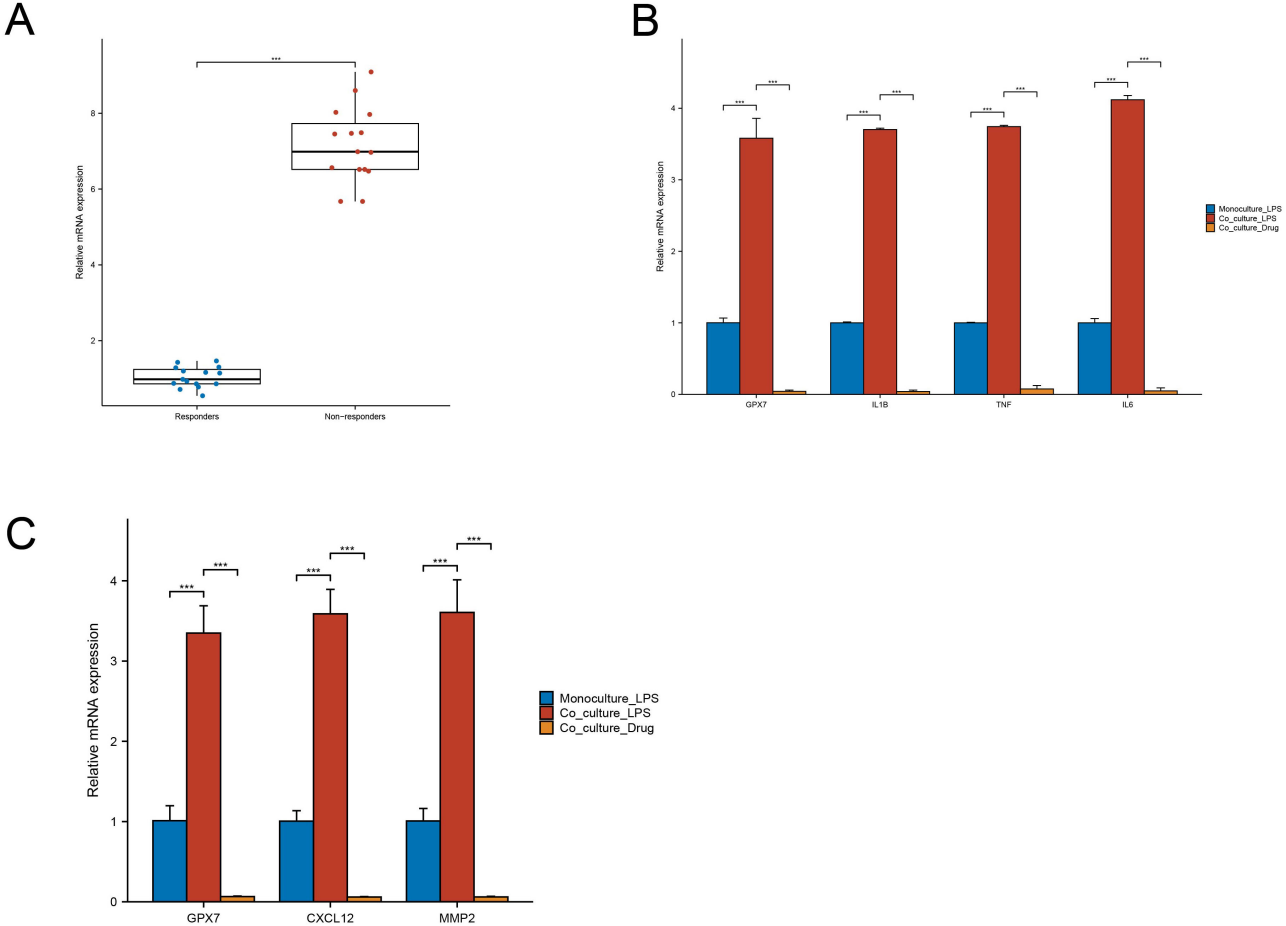
Clinical and co-culture qPCR validation of the GPX7-associated stromal–innate immune axis. **(A)** GPX7 mRNA expression measured by qPCR in colonic mucosal biopsies from UC patients stratified by IFX response, comparing IFX non-response and response groups. **(B)** Relative mRNA expression of GPX7, IL-1β, TNF, and IL6 in THP-1-derived macrophages under LPS-stimulated monoculture, LPS-stimulated fibroblast–macrophage co-culture, and LPS-stimulated co-culture treated with QL-X-138. **(C)** Relative mRNA expression of GPX7, CXCL12, and MMP2 in CCD-18Co fibroblasts under the same conditions. The co-culture condition amplified inflammatory and remodeling-associated transcriptional responses, whereas QL-X-138 attenuated these responses in both compartments.

In an independent IFX-stratified UC cohort, GPX7 mRNA expression was significantly elevated in IFX non-response samples compared with responders (**Figure 10A**). Using a CCD-18Co/THP-1 Transwell co-culture system, we found that LPS stimulation enhanced macrophage inflammatory gene expression (GPX7, IL-1β, TNF, and IL6; **Figure 10B**) and fibroblast remodeling-associated gene expression (GPX7, CXCL12, and MMP2; **Figure 10C**) relative to the corresponding monoculture condition. QL-X-138 attenuated these responses in both compartments, supporting pharmacologic modulation of the GPX7-associated stromal–innate immune axis.

## 4 Discussion

Our study uses a genetically informed (37, 38), multi-modal framework to interrogate SGOC-linked biology in UC through the lens of molecular innate immunity. By integrating cis-eQTL MR, independent replication, cross-cohort mucosal transcriptomics, and single-cell localization, we move beyond association-driven biomarker discovery to define a mechanistically interpretable GPX7-centered axis that connects immunometabolic stress, innate inflammatory signaling, and stromal remodeling.

GPX7 provides a plausible mechanistic bridge between SGOC-adjacent redox control and innate immune-stromal remodeling. GPX7 is an endoplasmic reticulum resident peroxidase that uses hydrogen peroxide to support oxidative protein folding via protein disulfide isomerase oxidation (39). This function is relevant to inflammatory stromal states because activated fibroblasts operate under high secretory and oxidative stress while producing ECM, chemokines, and other mediators that shape innate immune cell recruitment. Within this setting, SGOC metabolism likely acts as an upstream enabler by supplying redox buffering capacity, methyl donors, and amino-acid intermediates needed for sustained inflammatory and matrix programs (11, 12). The recent demonstration that mitochondrial one-carbon metabolism is required for profibrotic glycine synthesis and collagen output in fibroblasts provides a direct precedent for this coupling (15).

Evidence linking SGOC biology to UC is beginning to emerge. Recent investigations have established a relationship between serine metabolism and epithelial and immune cell function in colitis (16). In addition, studies that analyze the transcriptome indicate that GPX7 may be implicated among UC-associated regulatory signals (40). Our study contributes to this literature by positioning GPX7 within a cell-resolving framework in which fibroblasts appear to integrate metabolic and inflammatory cross-talk. This interpretation aligns with recent syntheses on stromal biology highlighting that intestinal fibroblasts are more than just passive scaffolding structures, but actively engage in inflammatory fibrosis and in immune-cell communication (41–43).

A compact, interpretable gene axis could capture SGOC-relevant disease variation, despite the features selected not being canonical SGOC enzymes. The C2 subtype exhibited enhanced metabolism of one-carbon and transsulfuration pathways and ECM remodeling. Together, these findings support a higher-order SGOC-linked tissue state in which metabolic buffering, stromal remodeling, and immune composition co-vary across UC samples. Because the same subtype showed a heterogeneous immune and stromal composition, GPX7-centered SGOC biology is embedded in multicellular tissue organization rather than being a purely cell-autonomous signal (41, 42).

GPX7 was the feature most consistently supported across the analyses performed here. Its MR direction agreed with case-control expression patterns, and it retained cis-pQTL support under our filtering strategy. We therefore interpret GPX7 not as a proven causal driver or standalone biomarker, but as a genetically supported marker of a disease-relevant stromal–innate immune state.

This interpretation has the cellular context provided by our single-cell analyses. The upregulation of GPX7 became localized to cycling cells and fibroblasts; furthermore, within fibroblasts, GPX7 positivity identified a profibrotic program targeting glutathione metabolism and one-carbon/transsulfuration signatures. Fibroblasts with elevated GPX7 exhibited augmented levels of CXCL12 and MMP2, intensified signaling interactions, and enhanced activity of HIF1A. Together, these characteristics indicate that GPX7-high fibroblasts contribute to the maintenance of an innate inflammatory niche, rather than merely aiding in the formation of scar tissue.

The communication and perturbation analyses strengthen this model. Virtual knockout implicated ECM components, hypoxia/stress genes, and cytokine-related pathways downstream of GPX7, while CellChat inference suggested amplified fibroblast communication along ECM-receptor, growth-factor, and chemokine axes. Such a state is well positioned to couple tissue injury to recruitment and education of innate immune cells, thereby sustaining inflammatory-repair circuits in UC (43).

Beyond transcriptomics, the methylation-expression-disease triangulation adds mechanistic plausibility. Two-step MR mediation suggested that a substantial fraction of the methylation-UC association at cg12640469 may operate through GPX7 expression, with a comparatively smaller residual direct effect. Although MR-based mediation cannot fully exclude alternative causal structures, the result is compatible with a model in which epigenetic tuning contributes to stabilizing a GPX7-high inflammatory fibroblast state (18, 39).

Finally, our work extends from target nomination to computational prioritization of candidate modulators. Docking and MD simulations suggested that QL-X-138 may interact with GPX7 through a dynamically stabilized pocket involving TRP142, whereas Belinostat appeared less stable over the simulated timescale. These results should be interpreted as hypothesis-generating rather than as evidence of direct target engagement, but they provide a rational starting point for biochemical and cell-based follow-up.

Importantly, the newly added clinical and co-culture qPCR experiments provide initial experimental support for this model. Elevated GPX7 in IFX non-response biopsies extends the anti-TNF-associated signal to an independent clinical cohort. In the CCD-18Co/THP-1 Transwell system, LPS amplified inflammatory cytokine expression in macrophages and remodeling-associated gene expression in fibroblasts, whereas QL-X-138 attenuated both compartments. These findings support a GPX7-associated stromal–innate immune circuit in UC, although they do not yet establish direct GPX7 target engagement or cell-type-specific causality.

Several limitations remain. First, blood-derived cis-eQTL instruments may incompletely proxy colonic mucosal or fibroblast-specific regulation, and formal SMR/HEIDI colocalization was not performed; therefore, the MR results should be interpreted as genetic prioritization rather than definitive proof of causality. Second, the GPX7_POS fibroblast definition based on detectable expression may be affected by scRNA-seq dropout, and the single-cell atlas is modest in size and lacks spatial validation. Third, the IFX-stratified qPCR cohort was small and not powered for predictive biomarker modeling or multivariable confounder adjustment. Fourth, CCD-18Co fibroblasts and THP-1-derived macrophages are standardized cell-line models and may not recapitulate patient-derived UC stromal or myeloid states. Fifth, the docking, 50 ns MD, and MM/GBSA analyses are preliminary, short-timescale, single-trajectory structural simulations and do not establish direct GPX7 target engagement, specificity, cytotoxicity, or dose-response behavior. In addition, ACTB was used as a single qPCR reference gene, and future validation should include multiple reference genes with formal stability testing. Future work should validate GPX7 perturbation in primary human colonic fibroblasts and primary fibroblast–myeloid co-culture systems, with explicit measurement of chemokine output, redox stress, ECM secretion, and innate immune recruitment.

## Data Availability

The datasets presented in this study can be found in online repositories. The names of the repository/repositories and accession number(s) can be found in the article/**Supplementary Material**.

## Supplementary material

Supplementary Figure 1 scRNA-seq QC and cell-type annotation overview. **(A–C)** QC distributions (nFeature_RNA, nCount_RNA, percent.mt) by sample and by cluster; feature–count relationship. **(D)** tSNE showing Harmony-integrated clusters at resolution 0.5. **(E)** Marker dot plot supporting cell-type annotation.

Supplementary Figure 2 Molecular docking mode of the third candidate, QL-XII-47. **(A)** Global view of QL-XII-47 docked to GPX7. **(B)** Detailed 3D binding mode showing interactions with peripheral residues including GLN71, LYS42, and TYR43. **(C)** 2D interaction map.

Supplementary Figure 3 Detailed molecular dynamics trajectory analysis for the GPX7–Belinostat complex. Temporal evolution of key structural parameters over 50 ns. **(A)** Ligand RMSD. **(B)** Complex RMSD. **(C)** Protein RMSF. **(D)** Radius of Gyration (Rg). **(E, F) (E)** Number of intermolecular hydrogen bonds, and **(F)** Solvent Accessible Surface Area (SASA). The continuous fluctuations in Rg **(D)** and SASA **(F)** corroborate the unstable binding mode observed in the RMSD analysis.

Supplementary Figure 4 Detailed molecular dynamics trajectory analysis for the GPX7–QL-X-138 complex. Temporal evolution of key structural parameters over 50 ns. **(A)** Ligand RMSD. **(B)** Complex RMSD. **(C)** Protein RMSF. **(D)** Radius of Gyration (Rg). **(E, F) (E)** Number of intermolecular hydrogen bonds, and **(F)** Solvent Accessible Surface Area (SASA). Note that after the initial conformational shift (~10 ns), the Radius of Gyration **(D)** stabilizes and remains flat, confirming that the complex reaches a highly stable, compact state following the induced-fit adjustment.
