# Supplementary figures and images for: GPX7 marks fibroblast-associated stromal–innate immune crosstalk in ulcerative colitis
Source: Front Immunol. 2026 May 22;17:1856998. doi: 10.3389/fimmu.2026.1856998 (PMC13236626; doi:10.3389/fimmu.2026.1856998)

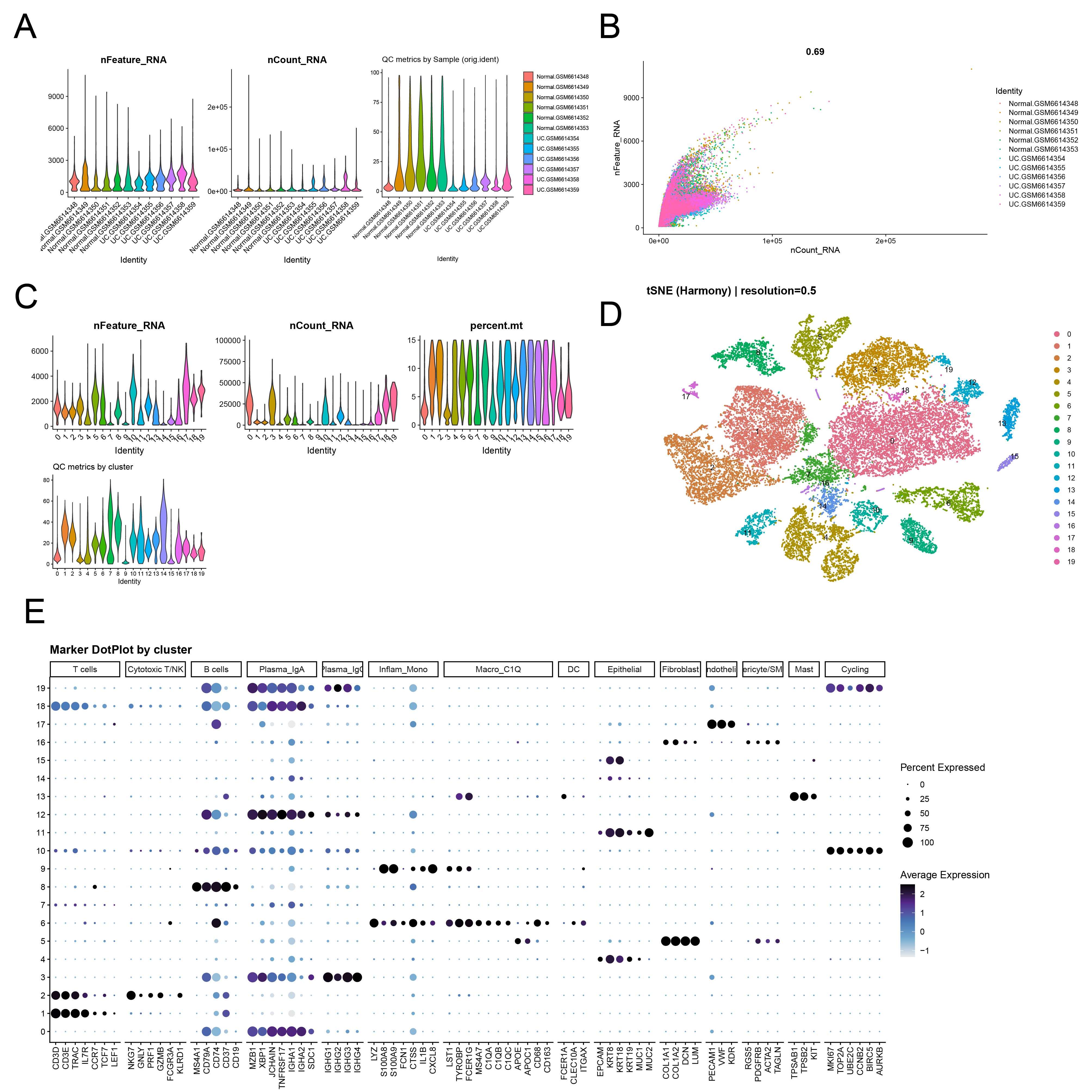

Supplement: Supplementary Figure 1 — scRNA-seq QC and cell-type annotation overview. (A–C) QC distributions (nFeature_RNA, nCount_RNA, percent.mt) by sample and by cluster; feature–count relationship. (D) tSNE showing Harmony-integrated clusters at resolution 0.5. (E) Marker dot plot supporting cell-type annotation. [file Image1.jpeg]

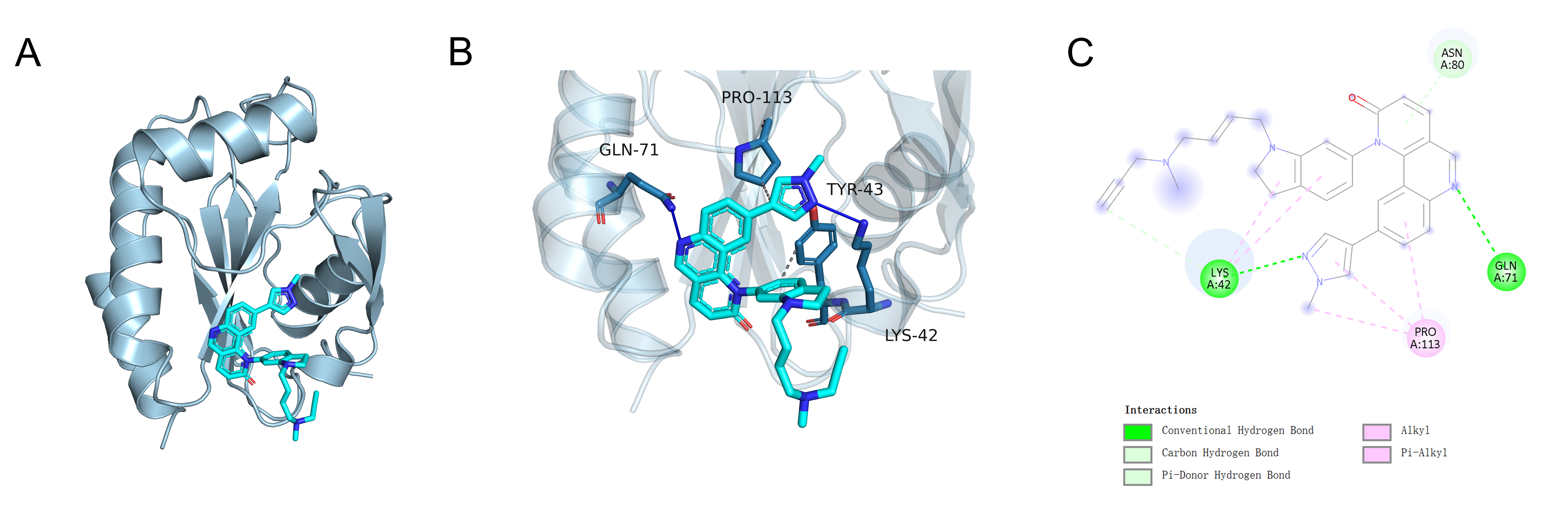

Supplement: Supplementary Figure 2 — Molecular docking mode of the third candidate, QL-XII-47. (A) Global view of QL-XII-47 docked to GPX7. (B) Detailed 3D binding mode showing interactions with peripheral residues including GLN71, LYS42, and TYR43. (C) 2D interaction map. [file Image2.tif]

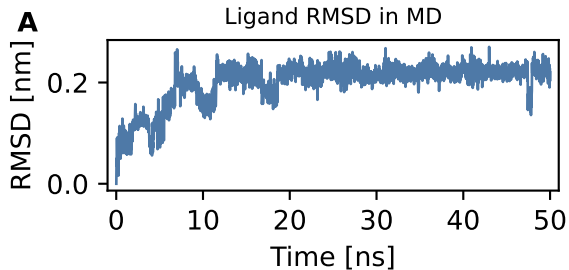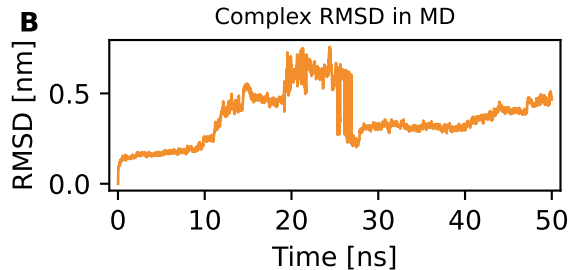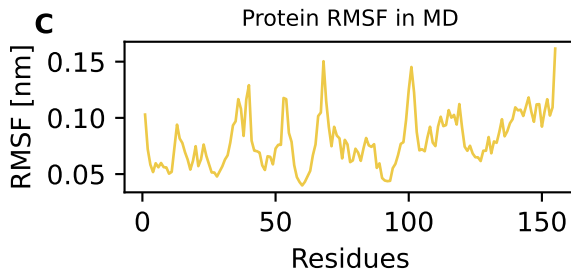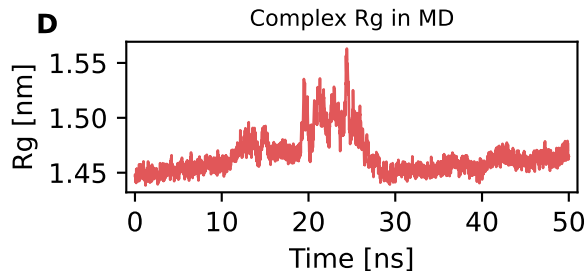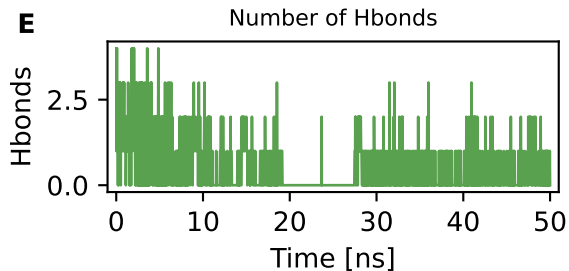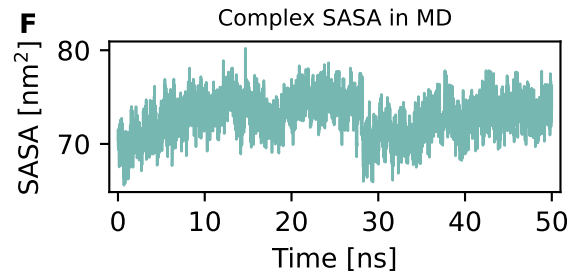

Supplement: Supplementary Figure 3 — Detailed molecular dynamics trajectory analysis for the GPX7–Belinostat complex. Temporal evolution of key structural parameters over 50 ns. (A) Ligand RMSD. (B) Complex RMSD. (C) Protein RMSF. (D) Radius of Gyration (Rg). (E, F) (E) Number of intermolecular hydrogen bonds, and (F) Solvent Accessible Surface Area (SASA). The continuous fluctuations in Rg (D) and SASA (F) corroborate the unstable binding mode observed in the RMSD analysis. [file DataSheet1.pdf]

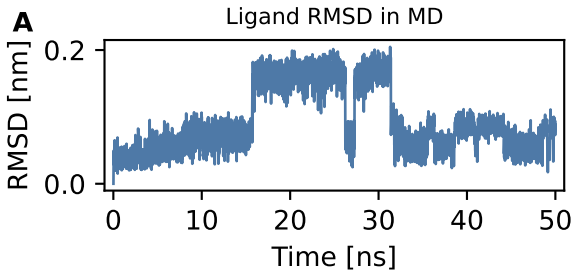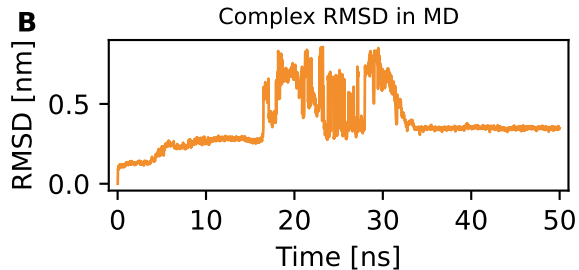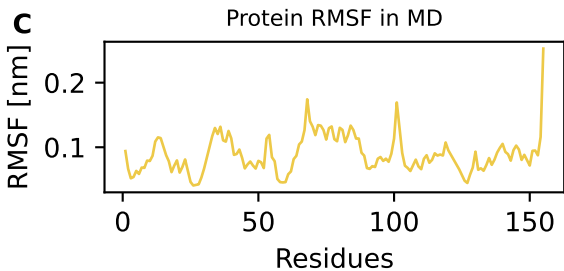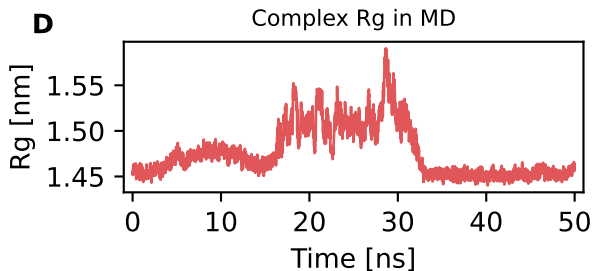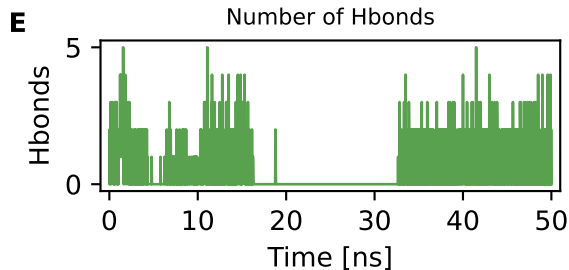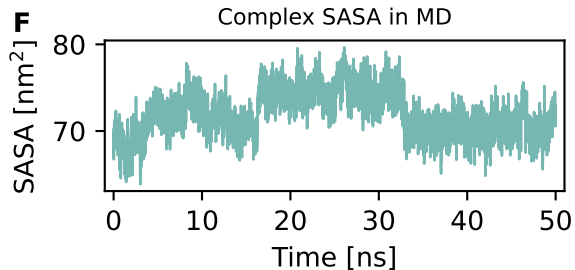

Supplement: Supplementary Figure 4 — Detailed molecular dynamics trajectory analysis for the GPX7–QL-X-138 complex. Temporal evolution of key structural parameters over 50 ns. (A) Ligand RMSD. (B) Complex RMSD. (C) Protein RMSF. (D) Radius of Gyration (Rg). (E, F) (E) Number of intermolecular hydrogen bonds, and (F) Solvent Accessible Surface Area (SASA). Note that after the initial conformational shift (~10 ns), the Radius of Gyration (D) stabilizes and remains flat, confirming that the complex reaches a highly stable, compact state following the induced-fit adjustment. [file DataSheet2.pdf]
